# MeCP2 deficiency exacerbates the neuroinflammatory setting and autoreactive response during an autoimmune challenge

**DOI:** 10.1038/s41598-021-90517-8

**Published:** 2021-05-26

**Authors:** M. I. Zalosnik, M. C. Fabio, M. L. Bertoldi, C. N. Castañares, A. L. Degano

**Affiliations:** 1 Departamento de Química Biológica Ranwel Caputto, Facultad de Ciencias Químicas, Universidad Nacional de Córdoba, X5000HUA Córdoba, Argentina; 2 Centro de Investigaciones en Química Biológica de Córdoba, Consejo Nacional de Investigaciones Científicas y Técnicas (CIQUIBIC, CONICET), Universidad Nacional de Córdoba, X5000HUA Córdoba, Argentina; 3 Instituto de Investigación Médica Mercedes y Martín Ferreyra, Consejo Nacional de Investigaciones Científicas y Técnicas, Universidad Nacional de Córdoba (INIMEC-CONICET-UNC), Córdoba, Argentina

**Keywords:** Autoimmunity, Immunological disorders, Inflammation, Neuroimmunology, Neurological disorders

## Abstract

Rett syndrome is a severe and progressive neurological disorder linked to mutations in the MeCP2 gene. It has been suggested that immune alterations may play an active role in the generation and/or maintenance of RTT phenotypes. However, there is no clear consensus about which pathways are regulated in vivo by MeCP2 in the context of immune activation. In the present work we set to characterize the role of MeCP2 during the progression of Experimental Autoimmune Encephalomyelitis (EAE) using the MeCP2^308/y^ mouse model (MUT), which represents a condition of “MeCP2 function deficiency”. Our results showed that MeCP2 deficiency increased the susceptibility to develop EAE, along with a defective induction of anti-inflammatory responses and an exacerbated MOG-specific IFNγ expression in immune sites. In MUT-EAE spinal cord, we found a chronic increase in pro-inflammatory cytokines gene expression (IFNγ, TNFα and IL-1β) and downregulation of genes involved in immune regulation (IL-10, FoxP3 and CX3CR1). Moreover, our results indicate that MeCP2 acts intrinsically upon immune activation, affecting neuroimmune homeostasis by regulating the pro-inflammatory/anti-inflammatory balance in vivo. These results are relevant to identify the potential consequences of MeCP2 mutations on immune homeostasis and to explore novel therapeutic strategies for MeCP2-related disorders.

## Introduction

Methyl-CpG binding protein 2 (MeCP2) is an X-chromosome nuclear protein that recognizes and binds specifically to methylated cytosine residues in the DNA, in regions enriched with adjacent A/T bases^1^. MeCP2 has been reported to play multifunctional roles in regulating gene expression, participating in RNA splicing^2^, chromatin remodeling^3,4^**,** transcriptional activation^5^ and repression^6^. This protein is expressed in all tissues although it is particularly abundant in the central nervous system (CNS)^7^. Since loss-of-function mutations in the MECP2 locus is the etiological cause of 95% of typical Rett syndrome^5,8^, MeCP2 role in the CNS has been extensively studied. Rett syndrome (RTT, MIM 312750) is one of the most common causes of mental retardation in females, with an incidence of 1 in 10,000–15,000^8^. Comprehensive analyses have revealed that early in development, classical RTT patients present with subtle, but consistent impairments that precede the onset of more obvious symptoms, which include lack or gradual loss of speech and purposeful hand use, stereotypic hand movements, seizures, autistic-like features, ataxia, and breathing disturbances^9^.

Although most studies have explored the critical role of MeCP2 in neural function, MeCP2 expression is ubiquitous and the pathogenesis of RTT compromise other systems besides the CNS^10^. In this regard, polymorphisms in *MECP2* have been linked to increased susceptibility to autoimmune diseases in humans, such as systemic lupus erythematosus (SLE)^11,12^, thyroid diseases^13^ and primary Sjogren’s syndrome^14^. MeCP2 is expressed in immune cells, and alterations in MeCP2 expression levels affect immune function and cytokine production^15–20^. More recently, a wealth of evidence indicates that redox imbalance, mitochondrial dysfunction, immune alterations and systemic subclinical inflammation are crucial players in a process coined “oxInflammation” that strongly affects clinical progression in RTT patients^21–27^.

All these data suggest that *Mecp2* mutations may affect the immune homeostasis and thus, it is reasonable to consider that the immune system may play an active role in the generation and/or maintenance of RTT alterations. Still, there is no clear consensus about which pathways or genes are regulated in vivo by MeCP2 in the context of immune activation. Another limitation of previous studies, is that they used “null” mouse models, with total absence of MeCP2 protein. From a potentially translational point of view, the interpretation of these results is complex in the context of RTT and associated disorders, in which MeCP2 protein is indeed expressed, although at lower levels or as a functionally deficient protein.

Thus, in the present work we set to explore the role of MeCP2 during the progression of Experimental Autoimmune Encephalomyelitis (EAE), considering that this experimental model provides the opportunity to characterize the autoimmune response and concomitant neuroinflammatory status. We used a well characterized RTT mouse model (MeCP2^308/y^), that carries an early termination codon and generates an MeCP2 protein truncated at amino acid 308, with loss of the C-terminal region^28^. This truncated form of MeCP2 lacks several regulatory sites, such as phosphorylation sites that are key for co-repressors/co-activators binding^29,30^. Likewise, the absence of the C-terminal domain leads to lower stability and shorter half-life of the truncated protein compared to the wild-type (WT) form^31^. As a consequence, this model represents a condition of “MeCP2 function deficiency”, as opposed to “total MeCP2 absence” represented by *Mecp2*-null models. The MeCP2^308/y^ mutant model shows a progressive neurological phenotype and longer lifespan, making it more suitable for immune studies and EAE induction. In this sense, although MeCP2^308/+^ mutated female could be the ideal model for translational implications, in the present work we decided to characterize the response in MeCP2^308/y^ mutant males in order to minimize animal use and variability derived from random X-inactivation.

Our results showed that MeCP2 deficiency increased the susceptibility to develop EAE, along with an exacerbated inflammatory profile and a defective induction of anti-inflammatory responses (both in peripheral immune sites and in the CNS). Importantly, in the absence of immune activation, we detected no differences between WT and MeCP2^308/y^ in any of the parameters tested. To our knowledge, this is the first report that explored the role of MeCP2 in the pathophysiology and neuroinflammatory response in the context of an autoimmune challenge. Our results suggest that MeCP2 acts intrinsically upon immune activation, affecting neuroimmune homeostasis by regulating the pro-inflammatory/anti-inflammatory balance in vivo.

## Results

### Mecp2^308/y^ mice show increased susceptibility to develop EAE

In order to evaluate the role of MeCP2 during an immune challenge in vivo, EAE was induced in WT and MUT mice by injection of MOG/CFA mix or CFA alone as control, and clinical signs were evaluated daily for 30 days. During acute EAE (typically ~ 12 days post induction, dpi) animals showed the most severe symptoms characterized by tail and hind limb paralysis, followed by partial recovery; during the chronic phase (> 20 dpi) mice presented permanent residual motor deficits^32^. All MeCP2-MUT mice injected with MOG developed EAE clinical signs; in contrast, only 84% of WT mice develop EAE (WT mice that did not develop the disease were discarded from subsequent analyses). None of the mice died as a result of EAE. In addition, none of the animals treated with CFA alone developed the disease or showed motor abnormalities after CFA injection (WT-CFA n = 7; MT-CFA = 8—data not shown).

**Figure 1:**
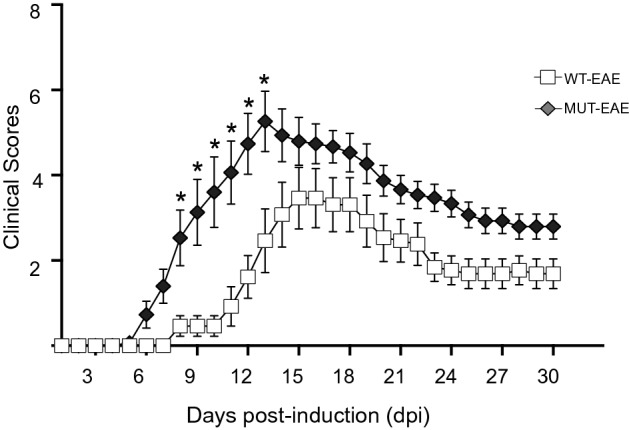
Clinical course of EAE. WT and MeCP2 mutant mice (MUT) were immunized with MOG and clinical signs were evaluated daily for 30 days. MUT-EAE mice showed an early EAE onset and developed more severe clinical signs in comparison to WT littermates. Data represent EAE clinical scores per group (mean ± SEM). Clinical scores per animal were calculated by adding tail and hind legs scores, and establishing a scale from 0 to 8, where 8 represents the highest EAE score per animal (i.e. 2 for the maximum tail score and 3 the maximum for each leg). Tail abnormalities were graded as: 0, no deficits, 1 partial loss of tail tone, 2 total tail paralysis. Each hind limb was graded as: 0, normal gait; 1, mild hind limb weakness; 2, dragged limp with abnormal gait; 3, complete hind limb paralysis with no residual movement. Two-factor ANOVA test with repeated measures was performed. *p < 0.05. WT-EAE n = 13; MUT-EAE n = 15.

Daily clinical assessment revealed significant differences in clinical scores between WT and MUT EAE groups (Two-factor ANOVA with repeated measures. Genotype × EAE, p = 0.0035). MUT-EAE mice showed an early onset of the disease and more severe clinical signs compared to WT-EAE (Fig. 1). In addition, during the chronic stage, MUT-EAE mice had a slower recovery of motor condition, but by 30 dpi both groups showed similar clinical scores. In order to further examine the severity in each experimental group, maximum clinical score (MSC) and disease index (DI) were calculated (see “Methods” section). No significant differences were found in the MCS between WT and MUT animals (data not shown); however, the accumulated disease index was significantly higher in MUT mice (WT-EAE 56.46 ± 8.72; MUT-EAE 89.87 ± 8.19; unpaired t-test two tailed p = 0.0111). These results suggest that MeCP2-MUT animals have a greater predisposition or vulnerability to develop EAE, as they showed an early onset of the disease, greater severity of clinical signs and required more time for partial recovery during the chronic phase.

### Splenocytes from Mecp2^308/y^ mice display a pro-inflammatory profile after MOG stimulation ex vivo

The initial immune response and maintenance of EAE occur mainly at the level of the lymphatic organs, where the antigen presentation triggers the activation and proliferation of effector cells^33^. Therefore, in order to understand the differences observed in EAE progression between WT and MUT animals, we set to examine the immune profile and specific response to MOG. To that end, splenocytes were isolated from WT and MUT animals either at the acute EAE phase (12 dpi) or at chronic stage (30 dpi), and they were re-stimulated with MOG in vitro (+ MOG). After 72 h of incubation, supernatants from each condition were isolated and concentrations of different cytokines (IFNγ, TNFα, IL-6, IL-17, IL-2, IL-4, IL-10 were determined using a specific kit for Th1/Th2/Th17 cytokines (as explained in “Methods” section).

#### Acute stage

**Figure 2:**
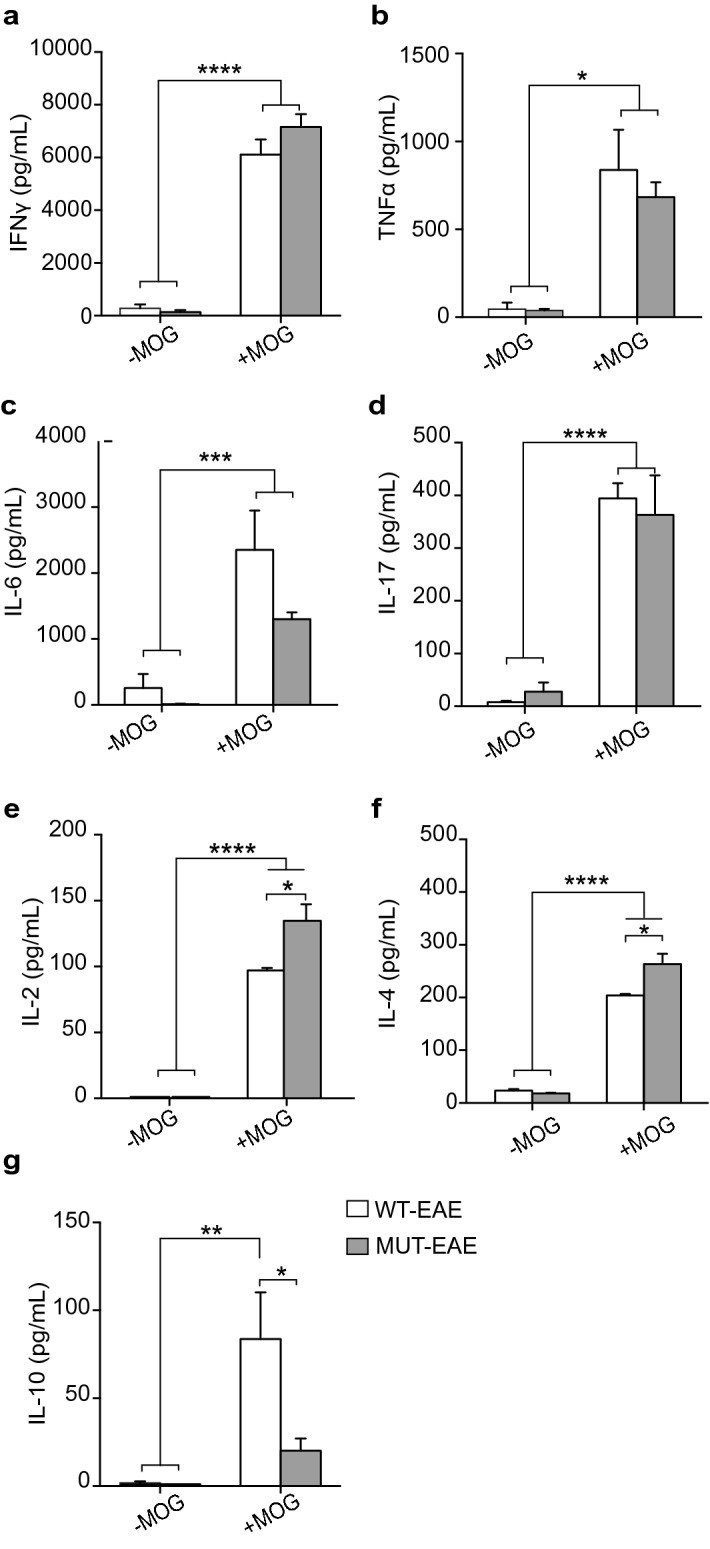
MOG-induced cytokine production during acute stage of EAE. Cytokines levels in supernatants from splenocytes isolated from WT-EAE and MUT-EAE animals was assessed in the absence or presence of MOG for 72 h using a cytometry kit (− MOG, + MOG, respectively). For all cytokines analyzed: (**a**) IFNγ, (**b**) TNFα, (**c**) IL-6, (**d**) IL-17, (**e**) IL-2, (**f**) IL-4, (**g**) IL-10, in vitro re-stimulation with MOG induced a significant increase in cytokine secretion in both WT and MUT groups, compared to the absence of MOG in culture (− MOG). (**e,f**) IL-2 and IL-4 levels secreted by MUT-EAE + MOG splenocytes were higher compared to WT-EAE + MOG, while IL-10 was only increased in WT-EAE (**g**). All values are presented as media ± SEM. Two-way ANOVA and Tukey post hoc test performed. *p < 0.05; **p < 0.002; ***p < 0.0002; ****p < 0.0001. WT-EAE n = 4; MUT-EAE n = 5.

We found no detectable levels of the tested cytokines in supernatants of splenocytes isolated from either control WT-CFA or MUT-CFA animals cultured with or without MOG (WT-CFA n = 4, MT-CFA n = 5). Similar high levels of MOG-induced pro-inflammatory cytokines (IFNγ, TNFα, IL-17 and IL-6) were secreted by WT-EAE and MUT-EAE splenocytes (Fig. 2a–d); however, MUT-EAE splenocytes + MOG, responded by increasing the production of IL-2 and IL-4, which was significantly higher than in WT-EAE + MOG group (Fig. 2e,f). On the other hand, we observed a lower production of IL-10 from MUT-EAE + MOG splenocytes compared to WT-EAE + MOG group (Fig. 2g).

**Figure 3:**
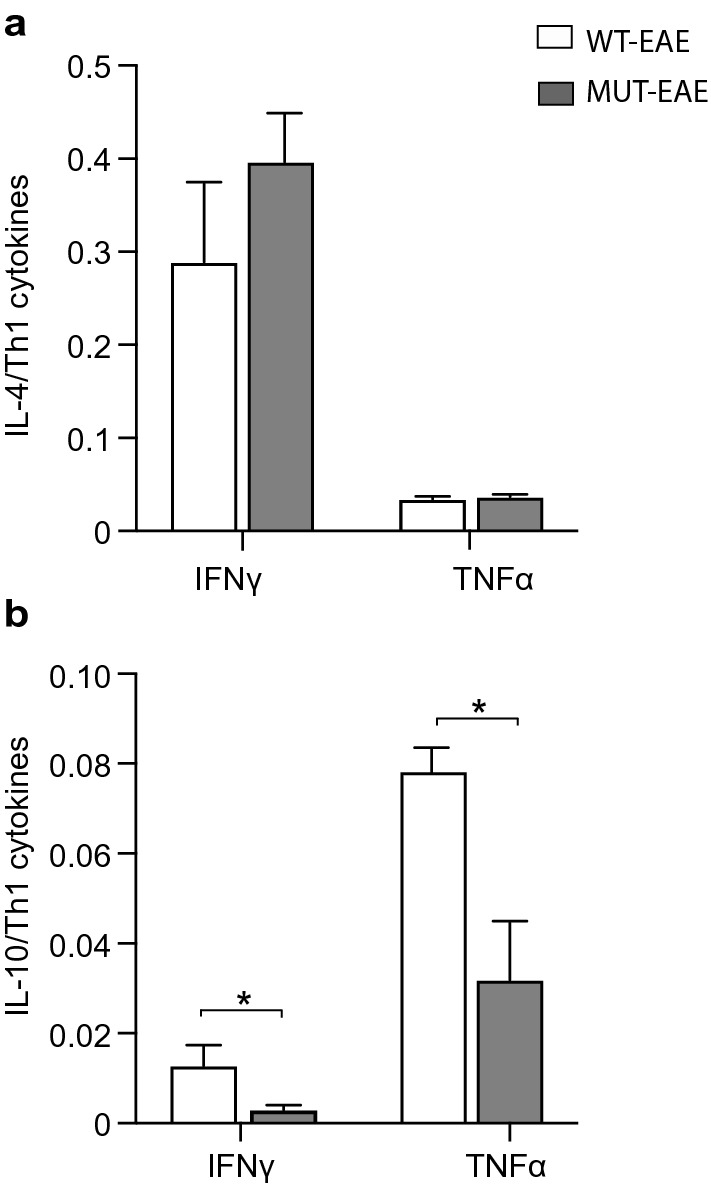
Th2/Th1 ratios during acute EAE. Comparative ratios were calculated between the production of anti-inflammatory cytokines (IL-4, IL-10) and the pro-inflammatory cytokines (IFNγ and TNFα) from WT-EAE and MUT-EAE isolated splenocytes re-stimulated in vitro with MOG. The ratios between (**a**) IL-4/IFNγ and IL-4/ TNFα, (**b**) IL-10/IFNγ and IL-10/TNFα are displayed. The lower production of IL-10 from MUT-EAE + MOG splenocytes induced a decrease in the Th2/Th1 balance during the acute stage. All values are presented as media ± SEM. Mann–Whitney non-parametric test was performed. *p < 0.05. WT-EAE n = 4; MUT-EAE n = 5.

Considering that the progression of immune response along EAE clinical course, depends on the balance of pro- and anti-inflammatory cytokines, we calculated Th2/Th1 ratios between measured concentrations of anti-inflammatory cytokines (IL-4 and IL-10) and pro-inflammatory cytokines (IFNγ and TNFα) secreted by WT-EAE and MUT-EAE splenocytes in response to MOG (Fig. 3). No significant differences were found in IL-4/TNFα or IL-4/IFNγ ratios between WT and MUT groups (Fig. 3a). However, both IL-10/IFNγ and IL-10/TNFα ratios were significantly lower compared to WT-EAE group (Fig. 3b). Thus, splenocytes from MUT-EAE mice during the acute phase, responded to MOG generating a skewed Th1 proinflammatory profile.

#### Chronic stage

**Figure 4:**
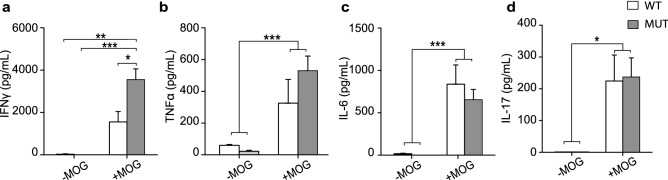
Cytokine production by splenocytes isolated during chronic stage of EAE. Concentration of cytokines produced by splenocytes from WT-EAE and MUT-EAE animals cultured for 72 h in the absence or presence of MOG (− MOG/+ MOG). For all cytokines analyzed (**a**) IFNγ, (**b**) TNFα, (**c**) IL-6, and (**d**) IL-17, MOG stimulation induced a higher and statistically significant production compared to unstimulated cells. Also, MUT splenocytes secreted significantly more IFNγ than WT splenocytes in response to MOG. All values are presented as media ± SEM. Two-way ANOVA and Tukey post hoc test were performed. *p < 0.05; **p < 0.002; ***p < 0.0002. WT-EAE n = 5; MUT-EAE n = 5.

**Figure 5:**
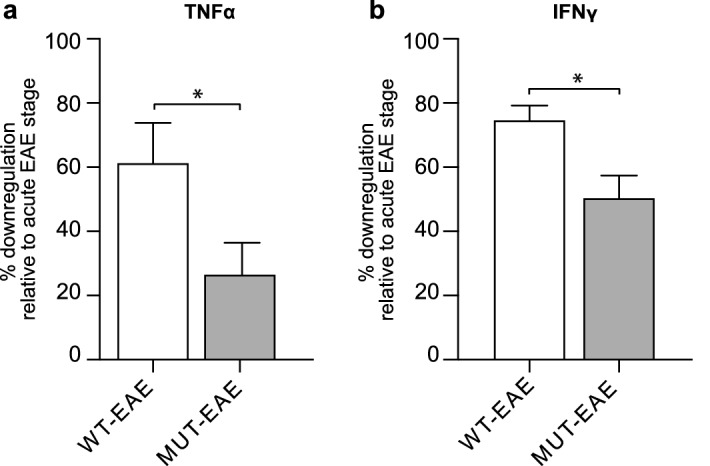
Downregulation of TNFα and IFNγ levels in chronic EAE. TNFα and IFNγ concentrations secreted by MOG-stimulated cells isolated from the acute phase were considered 100%. Using the concentrations secreted by MOG-stimulated cells isolated from the chronic phase, we calculated the percentage of TNFα and IFNγ levels secreted, relative to the acute phase. Data represents the percentage of downregulation, calculated as the difference between 100 and the relative percentage obtained at chronic phase per animal (mean ± SEM). Results were analyzed using an unpaired t-Student test. *p < 0.05. WT-EAE n = 4; MUT-EAE n = 5.

Concentration ranges for IL-2, IL-4 and IL-10 were below the detection limit of our kit, for all tested conditions. No significant levels of the tested cytokines were detected in supernatants from CFA splenocytes, irrespective of their genotype and re-stimulation with MOG (data not shown). Higher levels of TNFα, IFNγ, IL-6 and IL-17 were produced in MOG-stimulated groups compared with the controls (− MOG, Fig. 4a–d); only MOG-induced IFNγ production was significantly higher in MUT-EAE than in WT-EAE splenocytes (Fig. 4a). To further interpret cytokine regulation in MUT mice in the context of EAE progression, we calculated in what percent TNFα and IFNγ secretion were downregulated from acute (Fig. 2a,b) to chronic stage (Fig. 4a,b) for each group. MOG-induced IFNγ and TNFα secretion declined significantly more in WT-EAE cells than in MUT-EAE cells during the chronic phase (Fig. 5).

### Mecp2^308/y^ mice present more CNS infiltrating cells during acute EAE stages

Histological correlation of an autoimmune disease provides essential information on the underlying autoimmune pathology. Infiltration of T cells and mononuclear cells in the CNS is a key feature during EAE^34^. Therefore, we sought to assess whether MeCP2 deficiency could be influencing the infiltration of immune cells to the CNS, particularly at the level of the spinal cord, a target tissue in EAE. Histological analysis was performed on samples obtained during both acute and chronic phases of EAE. Lumbar spinal cord sections were stained with Toluidine Blue. In order to grade the level of cell infiltration, we established a scale by providing scores from the least severe (score 0, absence of infiltrates) to the most severe level of infiltration (score 3, as described in “Methods” section).

**Figure 6:**
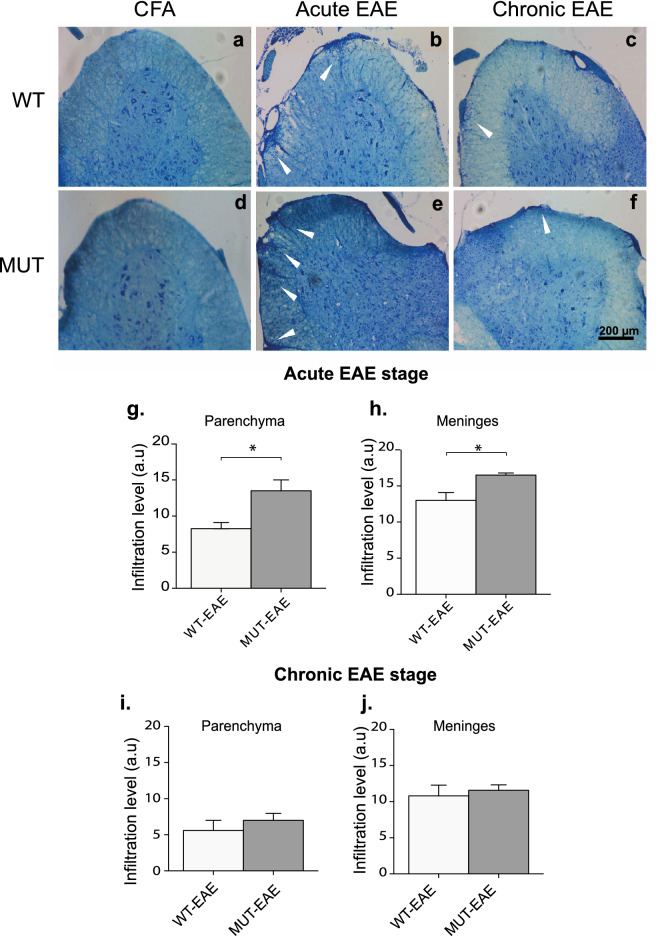
Analysis of infiltrating cells in spinal cord. (**a**–**f**) Cryosections of lumbar spinal cord were stained with Toluidine Blue and analyzed to determine the level of cellular infiltrates. Representative photos of coronal sections of spinal cords obtained from WT and MUT control animals (CFA) showed no cellular infiltrates (**a,d**). Representative spinal cord images from EAE mice showed typical cellular infiltrates (white arrows) in WT and MUT mice at acute (**b,e**) and chronic stages (**c,f**). Scale Bar: 200 μm. (**g–j**) The level of cellular infiltration in WT and MUT EAE groups was evaluated in meninges and parenchyma, as follows: 0, no infiltrates; 1, one or two cell focus; 2, more than 2 small focuses or one extensive focus; 3, more than two deep or extensive focus of cells. During acute stage, spinal cord from MUT-EAE animals showed higher level of infiltrates in both (**g**) parenchyma and (**h**) meninges compared to WT-EAE. In chronic EAE phase, the extents of cellular infiltration in both spinal cord compartments were lower respect to the acute phase; however, no significant differences were observed between genotypes at this stage (**i,j**). All values are presented as media ± SEM. Results were analyzed using an unpaired t-Student test. *p < 0.05. WT-EAE n = 5; MUT-EAE n = 5.

Sections from both WT and MUT animals treated only with CFA showed no infiltrates along the whole EAE course (Fig. 6a,d). In acute phase, lumbar spinal cord from EAE groups showed distinctive infiltration patterns (Fig. 6b,e; white arrowheads). However, MUT-EAE spinal cords showed significantly higher level of cell infiltrates in meninges and parenchyma compartments compared to WT-EAE group (Fig. 6g,h). The level of infiltration of immune cells in spinal cord decreased during chronic stage, and no significant differences were found between genotypes at that time point (Fig. 6c,f,i,j). These results suggest that MeCP2 mutation facilitates the infiltration of immune cells in the CNS during the acute EAE phase.

### Time-course of Iba1 expression and microgliosis after EAE induction in Mecp2^308/y^ mice

**Figure 7:**
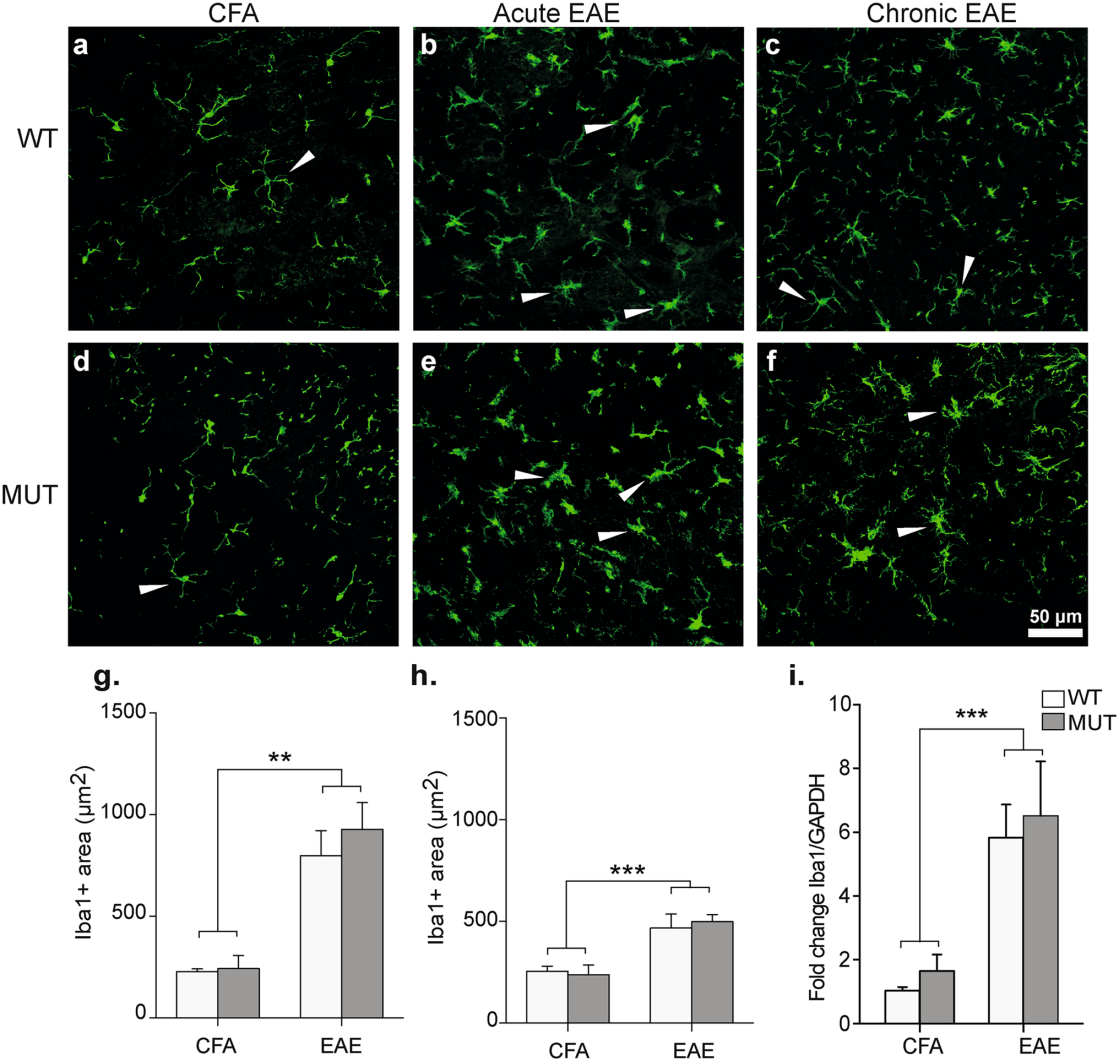
Microgliosis and Iba-1 expression analysis. (**a–f**) Representative sections of lumbar spinal cord from WT and MUT mice obtained at acute or chronic EAE stages and stained for the microglial marker Iba-1. Microglial cells from control CFA mice (**a**,**d**) showed small somas and thin projections. During acute EAE (**b,e**), microglia cell soma increased in size and projections became shorter and wider, indicative of microgliosis and microglial activation (white arrowheads). At chronic EAE stage (**c,f**) less cells evidenced an activated morphology (white arrowheads). Scale Bar: 50 μm. (**g,h**) Total area occupied by Iba-1 (µm^2^) was measured by a particle size exclusion analysis. In (**g**) acute and (**h**) chronic stages, Iba-1 + area was significantly higher in both WT-EAE and MUT-EAE group compared to CFA groups. Data is presented as mean ± SEM. A two-factor ANOVA test and Tukey post hoc test was performed. **p < 0.002, ***p < 0.0002. CFA-WT n = 4; CFA-MUT n = 4; EAE-WT n = 5; EAE-MUT n = 5, for each stage. (**i**) At 30 dpi, lumbar spinal cords were obtained from CFA and EAE mice in order to isolate the mRNA and analyze the expression levels of Iba-1 by Real Time RT-PCR. GAPDH was used as the housekeeping gene. Graph shows the fold change of expression calculated as 2^−ΔΔCT^. Iba-1 relative expression levels were higher in all mice immunized with MOG compared to CFA and it was similar between WT and MUT mice. All values are presented as mean ± SEM. Two-way ANOVA and Tukey post hoc test were performed. ***p < 0.0021. WT-CFA n = 6; MUT-CFA n = 8; WT-EAE n = 6; MUT-EAE n = 9.

Myeloid cells are key for the onset and progression of EAE. These cells adopt different phenotypes that coexist in the CNS after MOG immunization, and the balance between these phenotypes and the immune mediators they release are essential for mantainance of the chronic phase^35^. Since it has been shown that MeCP2 is expressed in microglial cells and that its deletion can alter the lifespan and pathogenic phenotype in mice^36^, we sought to evaluate the level of microgliosis after EAE induction. To this end, we performed immunostainings for Iba-1 in lumbar spinal cord from all groups and quantified the Iba-1^+^ area as indicator of microgliosis during acute and chronic EAE (Fig. 7a–f). At rest, microglial cells show a morphology characterized by long and thin processes extending from its soma, as observed in CFA control animals (Fig. 7a,d). After EAE induction (Fig. 7b,c,e,f), microglia becomes activated and fine processes get shorter and thicker, the cells suffer an increase in soma size, and those changes can be assessed as an increase in Iba-1^+^ area^37^. Our data revealed that Iba-1^+^ area (μm^2^) was significantly higher in EAE-induced mice (WT and MUT) along the course of the disease, in comparison to CFA groups (Fig. 7g,h). However, we found no significant differences between WT and MUT mice in any of the tested conditions and stages (i.e. CFA, acute or chronic EAE; Fig. 7g,h).

To further confirm if there were subtle changes in Iba-1 gene expression, we proceeded to evaluate the levels of spinal cord mRNA isolated from all groups. WT and MUT-EAE showed significant higher expression of Iba-1 transcript than CFA groups, regardless the genotypes (Fig. 7i). Similar results were obtained when we evaluated TSPO expression (translocator protein 18 kDa) another marker of gliosis in EAE^38^ (data not shown). Therefore, our results suggest that the deficit in MeCP2 would not affect the increased expression of Iba-1 and microgliosis in the context of EAE.

### Mecp2^308/y^ mice show higher and persisting inflammatory response in the CNS

**Figure 8:**
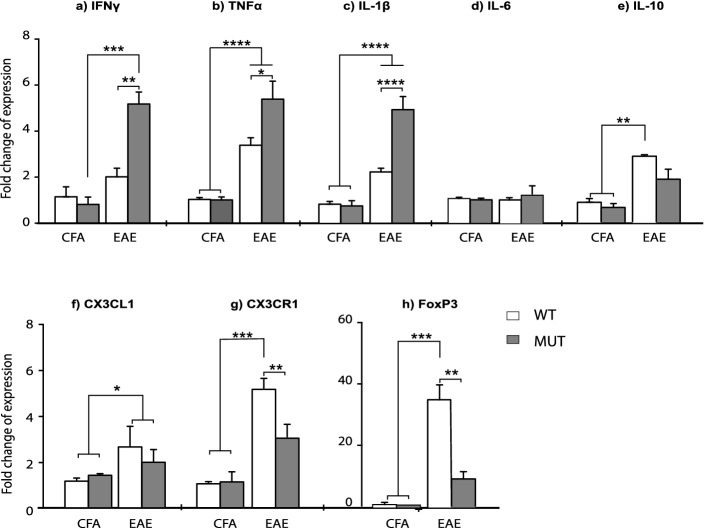
Gene expression of cytokines and immune mediators in spinal cords at chronic EAE. At 30 dpi, lumbar spinal cords were obtained from CFA and EAE mice in order to isolate mRNA and to analyze the levels of expression of the following: (**a**) IFNγ, (**b**) TNFα, (**c**) IL-1β, (**d**) IL-6, (**e**) IL-10, (**f**) CX3CL1, (g) CX3CR1 and (**h**) FoxP3, by Real Time RT-PCR. Graphs show the fold change of expression calculated as 2^−ΔΔCT^. GAPDH was used as the housekeeping gene for all cases. In spinal cords from MUT-EAE, relative gene expression of IFNγ, TNFα, and IL-1β (**a**–**c**) were significantly higher compared to WT-EAE. IL-10 expression (**e**) was upregulated in WT-EAE mice compared to both, WT-CFA and MUT-CFA groups. CX3CR1 and FoxP3 gene expression (**g,h**) were significantly lower in MUT-EAE mice compared to WT-EAE; in fact, no upregulation was observed in MUT-EAE compared to CFA groups. All values are presented as media ± SEM. Two-way ANOVA and Tukey post hoc test. *p < 0.05; **p < 0.001 ***p < 0.0001. WT-CFA n = 5; MUT-CFA n = 5; WT-EAE n = 6; MUT-EAE n = 6.

The effects of infiltrating cells on the CNS during the course of EAE depend on the release of cytokines and chemokines that are responsible for neuroinflammation^35^. In order to determine the inflammatory profile and regulatory environment in WT and MUT EAE animals, relative transcripts levels of several immune mediators were determined in the spinal cord during chronic EAE (Fig. 8). We found significant differences in IFNγ, TNFα, IL-1β and IL-10 mRNA expression levels between CFA and EAE groups (Fig. 8a–c,e); relative expression levels of these cytokines were significantly higher in the EAE group. Importantly, MUT-EAE animals displayed higher expression of IFNγ, TNFα and IL-1β compared to WT-EAE (Fig. 8a–c). In contrast to WT mice, MUT-EAE animals failed to show a significant increase of IL-10 mRNA respective to the control MUT-CFA (Fig. 8e).

Next, we assessed the relative expression of CX3CL1 chemokine and its receptor, CX3CR1. CX3CL1-CX3CR1 interaction mediates cell migration processes during EAE, impacting in the severity of the disease^39^. Although we found no significant differences in CX3CL1 expression between WT and MUT groups (Fig. 8f), the expression levels of the receptor (CX3CR1) in spinal cords from WT-EAE mice was significantly higher than in MUT-EAE (Fig. 8g). Lastly, we quantified the relative expression of FoxP3, the master gene for regulatory T cells. In WT animals, EAE induction led to an increased FoxP3 expression but in MUT animals, no significant differences were found between EAE and CFA mice (Fig. 8h).

## Discussion

Most basic research on MeCP2 and immunity have been performed in the absence of immune activation, using animal models with complete lack of MeCP2. In the present work, we proposed to characterize the immune profile elicited by Mecp2^308/y^ mice (MUT) in response to EAE induction, a prototypical experimental model of neuroinflammation. Our results showed that MeCP2 deficiency increased the susceptibility to develop EAE; MUT-EAE mice showed higher incidence and early onset of the disease (Fig. 1), which was accompanied by an increase of infiltrating cells in spinal cord during the acute stage (Fig. 6). We reasoned that an increase of infiltrating cells in MUT-EAE could be due to a higher proportion of autoreactive cells in these mice. Indeed, MUT-EAE splenocytes, isolated during the acute stage, responded to MOG by producing higher levels of IL-2 (Fig. 2e), a cytokine that induces the clonal expansion of T cells during EAE^40^. Thus, the proliferation of MOG-specific cells seems to be stimulated in a higher extent than in WT-EAE mice. In addition, splenocytes from MUT-EAE mice produced lower levels of MOG-induced IL-10, the classical immunoregulatory cytokine^41^ (Fig. 2g); moreover, we found that IL-10/IFNγ and IL-10/TNFα ratios were reduced compared to WT-EAE group (Fig. 3b), suggesting that MUT-EAE splenocytes generate a pro-inflammatory profile in response to MOG. At chronic stages, MOG-induced IFNγ production was significantly increased in MUT-EAE cells compared to WT-EAE cells (Fig. 4a). In this regard, a key feature of EAE chronic stage is that partial clinical remission is mediated by downregulation of pro-inflammatory response from immune cells^35^. Further analysis revealed that the downregulation of both TNFα and IFNγ expression from acute to chronic stages was also impaired in MUT-EAE splenocytes (Fig. 5). This setting resembles the immune features observed in IL-10-deficient mice^41^, suggesting that MeCP2 could regulate genes such as TNFα, IFNγ and/or IL-10, and in turn, in the absence of reciprocal regulation between these mediators, could exacerbate and maintain EAE pathogenesis. Altogether, our results suggest that MeCP2 deficiency accelerates the onset and increase the severity of EAE by skewing the response of MOG-specific cells towards a pro-inflammatory profile, at both acute and chronic stages of EAE.

It is noteworthy, that in the absence of MOG stimulation, we found no differences in cytokine expression between WT and MUT cells in culture (Figs. 2, 4). We propose that immune stimulation may be necessary to uncover differential immune responses in cultured MUT spleen cells. Our results are in agreement with previous work in which MeCP2-null macrophages expressed higher levels of inflammatory cytokines than WT macrophages, but only when they were stimulated with TNFα, since the same WT and MeCP2-null cells cultured without stimuli showed no differential response^19^. Interestingly, one study performed in human peripheral blood mononuclear cells (PBMC) made MeCP2 deficient with a lentiviral shRNA MeCP2 vector, displayed enhanced inflammatory cytokines expression, in the presence and absence of an inflammatory stimulus^18^. Also, Pecorelli et al. demonstrated that fibroblasts isolated from RTT patients showed that inflammasome machinery was already activated before stimulation^23^. Considering that these last studies were performed in primary cells isolated from humans, it is possible to consider that human cells were more likely to be exposed to immune stimulation than cells derived from mouse models kept under pathogen-free conditions in animal facilities. In any case, a growing number of evidence is supporting the concept that MeCP2 could function as a regulatory factor, preventing the perpetuation of an inflammatory profile.

It has been reported that microglial numbers and phagocytic function decreased gradually with symptoms progression in late-symptomatic Mecp2-null mouse model^36^. Conversely, in our study using Mecp2^308**/y**^ mouse model, we detected an increase of active microglia and similar levels of Iba-1 expression in both WT-EAE and MUT-EAE animals (Fig. 7g–i). Considering previous studies demonstrating that MeCP2-null microglia release five-fold higher levels of glutamate^42^, it is possible that MUT microglia also release glutamate in high levels, contributing to exacerbate glutamate excitotoxicity^43^, and to preserve the inflammatory status in the context of EAE. Also, we assessed whether other microglial markers could be affected in Mecp2^308**/y**^ mouse model. CX3CL1/CX3CR1 interaction mediates communication between neurons and microglia, and it is essential to modulate basic physiological activities during development and in pathological conditions^44^. Fractalkine (CX3CL1) is produced in neurons and endothelial cells^44^, and signals through the receptor CX3CR1, which is primarily expressed in microglia in the CNS^45^. In CX3CR1-KO mice, EAE induction showed higher severity associated with a greater accumulation of immune cells in the CNS^39,46^. In the present work, CX3CR1 expression increased significantly only in WT-EAE mice (Fig. 8f); therefore, the lower CX3CR1 expression detected in MUT-EAE mice may also contribute to maintain EAE symptoms in the chronic stage.

Considering that the immune profile in the CNS in situ modulates clinical progression in EAE, we also evaluated how different cytokines and immune markers are influenced by MeCP2 deficiency in spinal cord during chronic EAE. Regarding pro-inflammatory mediators, IL-1β is a critical mediator of EAE^47^ and TNFα production is associated with increased EAE severity, preventing the generation of an anti-inflammatory milieu needed for disease remission^48^. Here we showed that spinal cords from MUT-EAE mice displayed a persisting and higher inflammatory response in the CNS represented by increased levels of pro-inflammatory mediators IL-1β, TNFα and also IFNγ, when compared to WT-EAE (Fig. 8a–c). Interestingly, in the absence of neuroinflammatory challenge (CFA-injected animals), we did not observe significant differences in the expression of any of the cytokines tested between WT and MUT mice (Fig. 8a–h). In this sense, it has been shown that MeCP2 acts as a transcription factor that regulates neural development and connectivity in an activity-dependent manner^49^; therefore, it is likely that MeCP2 may act in an activity-dependent way in the context of immune responses.

Previous work has reported that, under basal conditions, several immune-related genes were expressed differentially in MeCP2 mouse models^19^; however, it is important to consider that most of those studies were performed using fully symptomatic MeCP2-308 mutated and *Mecp2-*null mouse models, or assessing different pathways, not tested here^19^. In addition, using different MeCP2-mutant murine models of RTT, it was demonstrated that oxidative brain damage was present at both pre- and symptomatic stages^26^, and basal subclinical inflammation was detected in plasma from symptomatic MeCP2-308 mutated females^50^. Since we have focused in assessing immune molecules in the context of an active immune challenge, it is possible that some of the reported inflammatory and oxidative stress markers are also affected at the younger ages tested in the present work (9–13 weeks old). Since animal age is crucial in MOG-induced EAE models^32^, we used 9 weeks-old WT and MeCP2 MUT mice in order to guarantee EAE susceptibility. This age has proven to work best in our hands, and during the experiment duration (4 weeks) no overt RTT phenotypes were observed in MeCP2 MUT mice, which simplified EAE clinical scoring. Considering that autoimmune responses and central immune tolerance are age-dependent processes, and MeCP2 MUT mice show a progressive developmental disorder, replicating these studies at later developmental stages might provide a more comprehensive understanding of neuroimmune interactions in the context of MeCP2 mutations.

Several authors have also reported significant alterations in immune markers and cytokines in blood, saliva and PBMC from RTT patients^16,18,20,23,51^. In those studies, several of the cytokines we evaluated in the present work showed different levels of variations (either up or downregulated) in samples from RTT patients in comparison with control subjects^16,17^. In this context, it is important to consider again, that most cohorts of RTT patients and age-matched controls are naturally exposed to immune stimulation, oxidative stressors, vaccinations and may rather represent our “stimulated conditions”, in which differential immune responses between genotypes become more obvious. We consider our findings are complementary to the existing studies that assessed immune, inflammatory and oxidative alterations in different RTT models, and we propose that in some scenarios, MeCP2 may functions as an activity-dependent regulator of immune function.

Finally, we had assessed the immune tolerance mediated by T regulatory cells expressing the transcription factor Forkhead box P3 (Treg FoxP3^+^) in WT and MUT mice. During EAE chronic stage, Treg FoxP3^+^ cells control the proliferation of autoreactive cells and the production of cytokines in the CNS^52–54^; and a higher frequency of Treg cells in the CNS correlates with EAE remission^55,56^. Interestingly, it has been reported that MeCP2 is required to maintain FoxP3 expression when Treg cells are activated^57^. We found similar results in Mecp2^308/y^ mice, and FoxP3 expression in MUT-EAE animals was significantly lower than in WT-EAE mice (Fig. 8g). Thus, a deficient MeCP2 regulation on Treg cells would explain in part the early onset of EAE and the presence of more severe clinical signs in MUT mice. Additionally, our results showed that MUT-EAE mice do not display a significant upregulation of IL-10 (Fig. 8e), a cytokine known to modulate Th17-associated pathogenesis in autoimmune diseases^58^. Thus, mutations in MeCP2 could be affecting also the release of regulatory cytokines, preserving a pro-inflammatory profile in the spinal cord.

**Figure 9:**
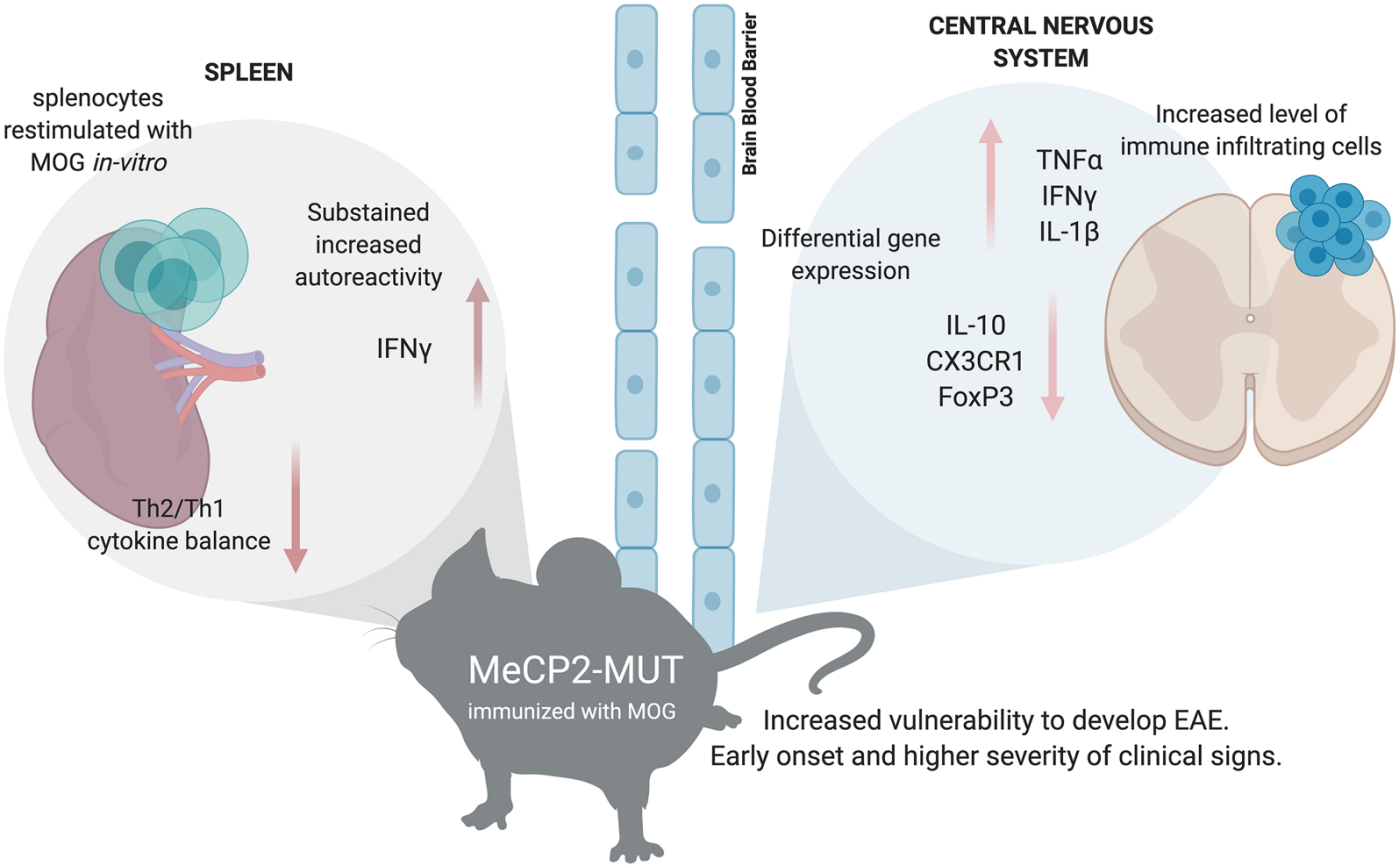
Schematic summary of the key neuroimmune features in Mecp2^308/y^ mice immunized with MOG. MeCP2 mutant mice (MUT) showed higher predisposition to develop EAE. Upon MOG immunization, these animals showed higher incidence, earlier onset of EAE and more severe clinical signs compared to WT mice. During acute EAE, increased levels of immune infiltrates were found in spinal cords in MUT mice. During the chronic stage, a neuroinflammatory profile persisted in these mice, demonstrated by higher gene expression of pro-inflammatory cytokines (IFNγ, TNFα and IL-1β) and lower expression of immunoregulatory mediators (IL-10, FoxP3 and CX3CR1) in spinal cord. Peripherally, splenocytes isolated from MUT-EAE mice showed exacerbated response to the autoantigen MOG, maintaining a sustained IFNγ response through the chronic EAE. These results highlight the essential role of MeCP2 in the regulation of proper immune responses and in maintaining neuroimmune homeostasis. Graph created with www.BioRender.com.

The present study proposed to evaluate in vivo in real time, the complexity of neuroimmunological interactions in the context of mutant MeCP2 protein (Fig. 9). MeCP2 deficiency increased the susceptibility to develop EAE, along with a defective induction of anti-inflammatory responses and an exacerbated IFNγ expression in immune sites. A chronic increase in gene expression of pro-inflammatory cytokines (IFNγ, TNFα and IL-1β) and downregulation of genes relevant for immune regulation (IL-10, FoxP3 and CX3CR1) was found in MUT-EAE spinal cords (Fig. 9). Future studies in MeCP2-308 mutated female mice, along with longitudinal studies of the immune response in the context of an autoimmune challenge will help to elucidate how MeCP2 influences the immune response along the lifespan.

## Conclusion

To our knowledge, this is the first report that explored in vivo and in real time the role of MeCP2 in the pathophysiology and neuroinflammatory response against an autoimmune challenge. Our results indicate that MeCP2 function is determined by immune activation, affecting neuroimmune homeostasis by regulating the pro-inflammatory/anti-inflammatory balance. These results are relevant to identify the potential consequences of MeCP2 mutations on immune homeostasis and to explore novel therapeutic strategies for MeCP2-related disorders.

## Methods

### Animals

For our studies we used the Mecp2^308/y^ mouse model (B6.129S-Mecp2/J, Stock 005439, The Jackson Labs). These animals carry a premature stop codon at amino acid 308, generating a truncated MeCP2 protein that lacks the C-terminal region^28^. The animals were kept at the facility of the “Departamento de Química Biológica-CIQUIBIC, Facultad de Ciencias Químicas” (Universidad Nacional de Córdoba, Argentina) and maintained in a C57BL/6J background. All the experiments were performed using only hemizygous MeCP2 males (MeCP2 MUT) and their corresponding WT male littermates as control, in order to avoid the high variability caused by random X-inactivation in females. All the animals were housed and maintained on a 12/12 h light/dark cycle (lights on at 7 am) with food and water *ad-libitum*. 2 weeks old mice were genotyped following the protocol provided by The Jackson Labs (https://www.jax.org/protocol/search) and then male littermates were divided into 4 different experimental groups: MeCP2-wildtype animals treated with complete Freund’s adjuvant (WT-CFA) or immunized with MOG (WT-EAE); MeCP2-mutant animals treated with complete Freund’s adjuvant (MUT-CFA) or immunized with MOG (MUT-EAE).

Animal procedures were fully reviewed and approved by our Institutional Animal Committee (IACUC-School of Chemistry, National University of Córdoba, Protocol 2015-832, renewed as 2019-2879), which follows guidelines from the National Institute of Health. We also confirm that all experiments were carried out in compliance with the ARRIVE guidelines (https://arriveguidelines.org).

### EAE induction

Male MeCP2 WT and MUT mice, 9 weeks old, were anesthetized via i.p. with a mixture of xylazine and ketamine (16 mg/kg and 80 mg/kg respectively) and immunized subcutaneously at the right and left flanks with 200 μl of an emulsion containing 200 μg of myelin oligodendrocyte glycoprotein peptide (MOG_35–55_, NH_2_-MEVGWYRSPFSRVVHLYRNGK-COOH; synthesized at the Johns Hopkins University Synthesis & Sequencing Core Facility, Baltimore, MD, USA). The peptide was dissolved in sterile water at 2 mg/ml, mixed at a 1:1 ratio with complete Freund’s adjuvant (CFA, Sigma-Aldrich Co., St. Louis, MO, USA), supplemented with 4 mg/ml of *Mycobacterium tuberculosis*. Pertussis toxin (200 ng List Labs, USA) was dissolved in 100 μl of phosphate-buffered saline (PBS) and injected i.p. the same day of the immunization and 48 h later^32^. Mice were scored daily for EAE symptoms starting at 6 days post induction (dpi). They were euthanized at either, 12 dpi (acute stage) or at 30 dpi (chronic stage). Given that the clinical signs of the tail and each leg did not develop together, we graded the signs for tail and hind legs separately and established a scale of 0–8, where the highest score 8 corresponds to the addition of a maximum tail score (2) and the maximum score for each leg (3). The tail abnormalities were scored as follows: 0, no deficits, (tail moves and can be raised, tail wraps around a round object if mouse is held at the base of the tail); 1 partial loss of tail tone, 2 total tail paralysis. Each hind limb was graded according to the following scale: 0, normal gait; 1, mild hind limb weakness; 2, dragged limp with abnormal gait; 3, complete hind limb paralysis with no residual movement. Importantly, MeCP2 308 mutant mice injected with CFA alone showed no differences in weight compared with WT, no obvious symptoms or motor abnormalities during the period tested (up to 30 dpi, i.e. 13 weeks old mice).

### Tissue processing

Animals were deeply anesthetized with an i.p. injection of ketamine-xylazine cocktail (16 mg/kg and 80 mg/kg respectively) and perfused intracardially with ice-cold PBS and then with ice-cold 4% paraformaldehyde in PBS. Spinal cords were removed and placed in 4% paraformaldehyde for 2 more hours. Then the tissues were buffered first, in 15% sucrose overnight and then in 30% sucrose solution for another 24 h at 4 °C. Finally, the tissues were embedded in mounting medium Cryoplast (Biopack, Buenos Aires, Argentina) and stored at − 80 °C until sectioning. 20 μm-thick serial sections of lumbar spinal cord were obtained using a cryostat (CM1510 S model, Leica Microsystems, Wetzlar, Germany), attached to aminopropyltriethoxysilane (APES, Sigma #A3648) treated-glass slides, and stored at − 80 °C until use.

### Toluidine blue staining

Six-step Sects. (20 µm) of lumbar spinal cords were incubated in toluidine blue 0.25% (ANEDRA, Tigre, Buenos Aires, Argentina) diluted in acid buffer (0.088 M acetic acid, 0.012 M sodium acetate) during 30 s and then washed in distilled water. Slides were left to dry and mounted using DPX (Sigma-Aldrich Co., St. Louis, MO, USA). Digital images were collected under optical microscope using a 10× and 20× objective. Slides were assessed in a blinded fashion for infiltrating immune cells in spinal cord in two different anatomical compartments (meninges and parenchyma). The level of infiltrating cells was scored for the meninges: 0, no infiltrates; 1, few isolated cells; 2 cell focuses; 3 extensive cell focuses. For parenchyma: 0, no infiltrates; 1, one or two cell focus; 2, more than 2 small focuses or one extensive focus; 3, more than two deep or extensive focus of cells^59^. For each animal, 6 serial coronal sections of lumbar spinal cords were analyzed and the infiltration level was obtained as the sum of the score’s infiltration in each of the sections.

### Immunofluorescence

Six-step serial coronal sections from lumbar spinal cords (20 µm) were blocked in Blocking Buffer (4% bovine fetal serum, 0.3% Triton in PBS) for 60 min at room temperature. Primary antibody (anti-Iba-1, 1:500, Abcam) was diluted in blocking buffer and incubated overnight at 4 °C in a humid light-tight box. Next, after 2 washes in PBS, the slides were incubated with the secondary antibody (Alexa 488-conjugated anti-rat IgG, 1:500) diluted in PBS for 1 h at room temperature. Afterwards, slides were incubated in DAPI for 5 min, washed 3 times in PBS and mounted using Mowiol 4–88 reagent (Aldrich, St. Louis, MO, USA). Images were acquired using a FV1000 confocal microscope (Olympus, Tokyo, Japan) equipped with argon/helium/neon lasers and 20× and 40× objectives. For each animal, 6 serial coronal sections of lumbar spinal cords were obtained and 5 fields per each coronal section was photographed in Z-projection using a 40× objective. The obtained images were subjected to a threshold and processed, to analyze the positive area of Iba expression (Iba-1^+^ area), using a size-based particle exclusion plugin (software ImageJ).

### Real time RT-PCR

Gene expression analysis was performed by real time RT-PCR. Mice were euthanized by cervical dislocation and lumbar spinal cords were removed. Tissues were homogenized and resuspended in 1 ml of TRIzol reagent (Invitrogen, Carlsbad, CA, USA) and then we proceeded according to manufacturer’s instructions for RNA extraction. 2 μg of RNA was incubated at room temperature for 15 min with DNase I (Invitrogen, Carlsbad, CA, USA). The product was incubated with random hexamer primers, deoxynucleotides and the reverse transcriptase M-MLV (Moloney Murine Leukemia Virus Reverse Transcriptase), all from Promega, Madison, WI, USA. Reverse transcription was performed following the manufacturer’s specifications, employing a thermocycler Mastercycler gradient (Eppendorf, Hamburg, Germany) in one cycle as follows: 6 min at 25 °C, 60 min at 37 °C, 18 min at 70 °C and 10 min at 4 °C. The generated cDNA was diluted with sterile milliQ water. For real time PCR reactions, 6 µl of cDNA was mixed with 0.375 µl of a 10 µM solution of each primer (sequences available upon request), 7.5 µl of 2 × SYBR Green PCR Master Mix (Promega, Madison, WI, USA) and sterile milliQ water to a final volume of 15 µl per tube. Duplicates were prepared for each sample. Real-time PCR was performed on the thermal cycler Rotor-Gene Q (Qiagen, Venlo, Limburg, Netherlands) according to the following protocol: Initial denaturation 10 min at 95 °C, amplification (45 cycles) with denaturation 15 s at 95 °C, annealing 30 s at 60 °C and extension 30 s at 70 °C. To confirm the presence of a single product, agarose electrophoresis and a melting curve of the DNA was always made covering the range of 50–95 °C. Relative gene expression levels were quantified using the comparative ΔΔCT method^60^. This method normalized CT values of the detected gene to the average of GAPDH and calculated the relative expression values as fold changes of the control group (WT-CFA), which was set at 1.

### Splenocytes culture and cytokine analysis

Control and EAE mice were euthanized 12 days post EAE induction (dpi; acute phase) or at 30 dpi (chronic phase). Under sterile conditions, the spleen was dissected and kept at 4 °C in sterile D-PBS with 2% endotoxin-free fetal bovine serum (FBS) and Gentamicin (50 μg/ml) until they were processed. After removing the fat, the spleen was disintegrated using a metal mesh. Subsequently, cells were incubated with 6 ml of red blood cell lysis buffer [Ammonium Chloride (NH4Cl) 0.15 M, Potassium Carbonate (KCO_3_) 10 mM, EDTANa_2_ 0.1 mM at pH 7.4] for 6 min and centrifugated for 10 min at 1600 rpm at 4 °C. The supernatant was discarded and the pellet was re-suspended with D-PBS supplemented with 10% FBS and Gentamicin, and centrifuged for 10 min at 1600 rpm at 4 °C. This last step was repeated twice. The precipitate containing the splenocytes was re-suspended in complete RPMI 1640 medium [10% FBS, Gentamicin (50 μg/ml), s-Glutamine (2 mM). The number and viability of cells were estimated by counting in 0.2% trypan blue solution in Neubauer chamber. Splenocytes concentration was adjusted to 1 × 10^6^ cells/ml, cultured by duplicate in complete RPMI medium and stimulated with MOG (1 μg/ml) or with complete medium alone, as negative control. Supernatants were collected at 72 h and levels of IFN-γ, TNF-α, IL-2, IL-4, IL-6, IL-10 and IL-17 were analyzed by triplicate per well, using the Cytometric Bead Array (CBA) Mouse Th1/Th2 Cytokine Kit (BD Biosciences) according to manufacturer’s instructions.

### Data analysis

The results are expressed as the mean ± SEM. Independent variables were analyzed using unpaired test t-student, or two-way analysis of variance (ANOVA). Whenever ANOVA indicated significant effects (p ≤ 0.05), a Tukey *Post-Hoc* test was carried out. In all cases, the assumptions of the analysis of variance (homogeneity of variance and normal distribution) were attained. In all statistical analysis, a p < 0.05 was considered to represent a significant difference between groups. All the analyses were performed using the software GraphPad Prism version 6.0 (La Jolla, California USA).

### Ethics approval and consent to participate

We declare that all animal procedures were fully reviewed and approved by the Institutional Animal Care and Use Committee, School of Chemistry, National University of Córdoba, Protocol number: 2015-832, renewed as 2019-2879, which follows guidelines from the National Institute of Health.

## Abbreviations

MeCP2: Methyl-CpG binding protein 2
RTT: Rett syndrome
Treg: Regulatory T cells
CFA: Complete Freund’s adjuvant
CNS: Central nervous system
dpi: Days post-induction
EAE: Experimental autoimmune encephalomyelitis
GAPDH: Glyceraldehyde-3-phosphate dehydrogenase
MOG: Myelin oligodendrocyte glycoprotein
MS: Multiple sclerosis
IFNγ: Gamma interferon
TNFα: Tumor necrosis factor
IL-6: Interleukin-6
IL-17: Interleukin-17
IL-2: Interleukin-2
IL-4: Interleukin-4
IL-10: Interleukin-10
FoxP3: Forkhead box P3
CX3CL1: Fractalkine
CX3CR1: Fractalkine receptor
